# Utility of GenBank and the Barcode of Life Data Systems (BOLD) for the identification of forensically important Diptera from Belgium and France

**DOI:** 10.3897/zookeys.365.6027

**Published:** 2013-12-30

**Authors:** Gontran Sonet, Kurt Jordaens, Yves Braet, Luc Bourguignon, Eréna Dupont, Thierry Backeljau, Marc De Meyer, Stijn Desmyter

**Affiliations:** 1 Royal Belgian Institute of Natural Sciences, OD Taxonomy and Phylogeny (JEMU), Vautierstraat 29, 1000 Brussels, Belgium; 2 Royal Museum for Central Africa, Department of Biology (JEMU), Leuvensesteenweg 13, 3080 Tervuren, Belgium; 3 University of Antwerp, Evolutionary Ecology Group, Groenenborgerlaan 171, 2020 Antwerp, Belgium; 4 National Institute of Criminalistics and Criminology, Vilvoordsesteenweg 100, 1120 Brussels, Belgium

**Keywords:** Forensic entomology, COI, DNA barcoding, BLAST

## Abstract

Fly larvae living on dead corpses can be used to estimate post-mortem intervals. The identification of these flies is decisive in forensic casework and can be facilitated by using DNA barcodes provided that a representative and comprehensive reference library of DNA barcodes is available.

We constructed a local (Belgium and France) reference library of 85 sequences of the COI DNA barcode fragment (mitochondrial cytochrome *c* oxidase subunit I gene), from 16 fly species of forensic interest (Calliphoridae, Muscidae, Fanniidae). This library was then used to evaluate the ability of two public libraries (GenBank and the Barcode of Life Data Systems – BOLD) to identify specimens from Belgian and French forensic cases. The public libraries indeed allow a correct identification of most specimens. Yet, some of the identifications remain ambiguous and some forensically important fly species are not, or insufficiently, represented in the reference libraries. Several search options offered by GenBank and BOLD can be used to further improve the identifications obtained from both libraries using DNA barcodes.

## Introduction

Insects collected on crime scenes can be used to estimate the time elapsed between death and corpse discovery, i.e. the post mortem interval or PMI (Rodriguez and Bass 1983, Joseph et al. 2011, Charabidze 2012). The correct identification of these insects is decisive in forensic casework since different species may have different developmental times under identical conditions. Erroneous identifications can therefore bias PMI estimates (Wells et al. 2001). DNA-based identification can be a valuable tool to identify immature life stages (Meiklejohn et al. 2013), fragments of insects, empty puparia (e.g. Mazzanti et al. 2010) or specimens of morphologically similar species (e.g. Meiklejohn et al. 2011, Jordaens et al. 2012). This technique relies on the comparison of a query sequence obtained from a sample collected at a crime scene with a library of reference sequences from well-identified specimens. The reference sequence showing the highest sequence similarity (= best match) with the query sequence can be used for its identification. However, the validity of this approach depends particularly on the reference library, which has to be representative, comprehensive and without misidentification or sequencing error (Wells and Stevens 2008).

In order to be of interest in court, species identifications provided by a specific reference library should be validated by assessing the likelihood of incorrect identifications using that library (Wells and Williams 2007, Wells and Stevens 2008). Sequences of a particular reference library may allow the correct identification of all species included in the library. However, if this library contains a limited set of species and ignores closely related species, then the likelihood of misidentifications is real (Wells and Stevens 2008). Moreover, the use of a reference library assembled in a different geographic area can also lead to incorrect species assignments because of geographic population structuring or eventual local hybrids (Stevens et al. 2002). Therefore, surveying local entomofaunas is a prerequisite for forensic specimen identifications (Vanin et al. 2008, Caine et al. 2009, Rolo et al. 2013). Likewise, assessing intraspecific variation and geographic substructuring is very important in forensic entomology (Wells and Williams 2007, Harvey et al. 2008, Desmyter and Gosselin 2009, Sonet et al. 2012).

The presence of pseudogene sequences and misidentified specimens in reference libraries is another problem that can constrain identification success (Wells and Stevens 2008). In order to minimise the risk of misidentifications caused by pseudogenes, an additional identification could be performed on the basis of an additional DNA fragment situated in another part of the mitochondrial genome (for example cytochrome *b*). Since most pseudogenes of mitochondrial origin are relatively short, the chance of sequencing two pseudogenes would drop substantially. Besides pseudogenes, sequences from misidentified specimens may be difficult to distinguish from haplotypes that are shared between correctly identified specimens from two different species (Whitworth et al. 2007). Increased sampling sometimes broadens the ranges of intra- and interspecific sequence divergences, even up to the point that they start overlapping so much that it becomes difficult to distinguish between the species (Wells et al. 2007).

Accurate identification of forensically important insects has been obtained using mitochondrial markers like the cytochrome *c* oxidase subunits I and II (COI and COII), cytochrome *b*, 16S rDNA, NADH dehydrogenase subunit 5, as well as nuclear markers like the ribosomal internal transcribed spacers 1 and 2, and the developmental gene bicoid (Sperling et al. 1994, Wells and Sperling 2000, Zehner et al. 2004, Guo et al. 2010, Li et al. 2010, Wang et al. 2010, Guo et al. 2011, Zaidi et al. 2011, Park et al. 2013). Among these markers, COI and COII have been predominantly used in forensic entomology (Sperling et al. 1994, Malgorn and Coquoz 1999, Vincent et al. 2000, Wallman and Donnellan 2001, Wells et al. 2001, Wells and Sperling 2001, Harvey et al. 2003, 2008, Wells and Stevens 2008, Liu et al. 2011, Boehme et al. 2012, Jordaens et al. 2012, Renaud et al. 2012). Coincidentally, a fragment of the 5’ end of COI has been selected as the standard barcode marker for animal identification by the Consortium for the Barcode of Life (Hebert et al. 2003). DNA barcodes are linked to voucher specimens and are associated with additional information such as primer data and trace files. This practice allows to verify the quality of sequences and to re-examine the organism from which the DNA was extracted (Ratnasingham and Hebert 2007). Barcodes are deposited in the Barcode of Life Data Systems (BOLD) and are tagged as barcodes in GenBank. Consequently, the 5’ end of COI is readily available in public reference libraries for a wide variety of dipterans of forensic interest (Wells and Stevens 2008).

In Western Europe, COI sequences from ca. 50 species of Sarcophagidae, ca. 10 species of Calliphoridae and five species of Muscidae are currently available as reference data for the identification of dipterans of forensic interest (Boehme et al. 2012, Jordaens et al. 2012). Specimens of seven species of Sarcophagidae and six species of Calliphoridae are from Belgium (Desmyter and Gosselin 2009, Jordaens et al. 2012, Marinho et al. 2012, Sonet et al. 2012). In this paper, we first extend the reference library of COI sequences with Belgian and French specimens of forensic interest belonging to two families (Calliphoridae and Muscidae) and secondly, we use these new sequences as queries to assess the validity of the identifications provided by GenBank and BOLD.

## Methods

### Specimens

We collected 85 adult specimens of 16 dipteran species of forensic interest from 24 localities in Belgium and three localities in France (Table 1). All Belgian specimens came from forensic cases. Three specimens from three species (*Neomyia cornicina*, *Polietes lardarius* and *Eudasyphora cyanella*) were collected on corpses but are currently not used for the calculation of the PMI. The French specimens of *Chrysomya albiceps* and *Lucilia sericata* were not collected on corpses, but were added because of their forensic interest. Morphological species identification was done by two taxonomic experts of Diptera (YB and ED), using five identification keys (D’Assis Fonseca 1968, Beĭ-Bienko 1988, Rozkošný et al. 1997, Gregor et al. 2002, Szpila 2012). Three *Fannia* specimens (Fanniidae) could not be identified to the species level and were considered as three putative different species. Specimens were deposited as vouchers at the National Institute of Criminalistics and Criminology in Brussels, Belgium (Table 1).

**Table 1.** Morphological identification and geographic origin of all specimens sampled in this study. Process ID of each sequence in the Barcode of Life Data Systems (BOLD) is given. For each haplotype (identified here by a number), the similarity of the best match (BM) retrieved from GenBank or BOLD using five search procedures is in italic if identification was ambiguous and normal font if identification was unambiguous. Ambiguous identifications where the best correct and the best incorrect matches had the same similarity with the query are indicated by an asterisk; best correct matches were more similar to the query than best incorrect matches in all other ambiguous identifications. No value is given when best matches were all of < 99% similarity and “na” when longer COI fragments were not available. Search procedures are: barcode fragment (642–658 bp) submitted to GenBank (1), to the public records of BOLD (2), to the species level records of BOLD including early releases (3), to the records of GenBank that are tagged as barcodes (4) and longer COI fragment (1412–1534 bp) submitted to GenBank (5).

Family	Species	Country	Locality	BOLD Process ID	Barcode fragment: haplotype ID	Longer COI fragment: haplotype ID	Similarity with BM in procedures 1 & 2 (%)	Similarity with BM in procedure 3 (%)	Similarity with BM in procedure 4 (%)	Similarity with BM in procedure 5 (%)
Calliphoridae	*Calliphora vicina* Robineau-Desvoidy, 1830	Belgium	Saint–Gilles/Sint–Gillis	NICC001-13	1	1	99.72*	99.83*	99.54	99.03
		Belgium	Schaerbeek/Schaarbeek	NICC002-13	2	na	100*	100*	99.85	na
		Belgium	Andrimont	NICC003-13	2	2	100*	100*	99.85	99.05
		Belgium	Hastière	NICC004-13	2	3	100*	100*	99.85	99.23
		Belgium	Humbeek	NICC005-13	2	4	100*	100*	99.85	99.16
		Belgium	Ixelles/Elsene	NICC006-13	3	5	99.72*	99.83*	99.54	
		Belgium	Auderghem/Oudergem	NICC007-13	2	3	100*	100*	99.85	99.23
		Belgium	Lier	NICC008-13	4	na	100*	100*	99.85	na
		Belgium	Gent	NICC009-13	5	6	99.86*	100*	99.7	
		Belgium	Saintes	NICC010-13	6	7	99.86*	99.6*	99.7	99.1
		Belgium	Bruxelles/Brussel	NICC011-13	2	3	100*	100*	99.85	99.23
		Belgium	Hastière	NICC012-13	7	8	99.86*	99.86*	99.7	99.03
		Belgium	Auderghem/Oudergem	NICC013-13	8	9	100*	100*	100	99.1
		Belgium	Schaerbeek/Schaarbeek	NICC014-13	9	10	99.72*	99.83*	99.54	99.03
	*Calliphora vomitoria* (Linnaeus, 1758)	Belgium	Toernich	NICC015-13	10	na	100	100	99.09	na
		Belgium	Hastière	NICC016-13	10	11	100	100	99.09	99.02
		Belgium	Andrimont	NICC017-13	11	12	99.58	100	99.38	
		Belgium	Schaerbeek/Schaarbeek	NICC018-13	10	11	100	100	99.09	99.02
		Belgium	Genk	NICC019-13	10	11	100	100	99.09	99.02
		Belgium	Laeken/Laken	NICC020-13	10	13	100	100	99.09	
		Belgium	Liège	NICC021-13	10	11	100	100	99.09	99.02
		Belgium	Saintes	NICC022-13	10	14	100	100	99.09	
		Belgium	Steendorp	NICC023-13	10	11	100	100	99.09	99.02
		Belgium	Gent	NICC024-13	10	11	100	100	99.09	99.02
		Belgium	Hastière	NICC025-13	10	na	100	100	99.09	na
		Belgium	Schaerbeek/Schaarbeek	NICC026-13	10	11	100	100	99.09	99.02
		Belgium	Genk	NICC027-13	10	11	100	100	99.09	99.02
		Belgium	Sint–Laureins	NICC028-13	10	11	100	100	99.09	99.02
		Belgium	Schoonaarde	NICC029-13	12	15	99.86	100	98.94	
		Belgium	Antwerpen	NICC030-13	10	na	100	100	99.09	na
	*Chrysomya albiceps* (Wiedemann, 1819)	France	St Pourçain/Sioule	NICC031-13	13	16	100	100		99.29
		France	St Pourçain/Sioule	NICC032-13	14	na	100	100		na
		France	St Pourçain/Sioule	NICC033-13	15	17	99.86	100		99.23
		France	St Pourçain/Sioule	NICC034-13	13	16	100	100		99.29
		France	St Pourçain/Sioule	NICC035-13	14	na	100	100		na
		France	Sarreguemines	NICC036-13	13	16	100	100		99.29
		Belgium	Meerdaalwoud	NICC037-13	16	na	99.86	100		na
	*Cynomya mortuorum* (Linnaeus, 1761)	Belgium		NICC038-13	17	18	99.73	100		
	*Lucilia ampullacea* Villeneuve, 1922	Belgium	Flémalle	LUCIL001-12	18	19	100	100		99.23
		Belgium	Flémalle	LUCIL002-12	19	20	99.86	99.86		99.1
	*Lucilia sericata* (Meigen, 1826)	Belgium	Hastière	LUCIL061-12	20	21	99.86	100	100	99.23
		Belgium	Auderghem/Oudergem	LUCIL062-12	21	na	100	100*	100	na
		Belgium	Gent	LUCIL063-12	22	22	100	100*	100	99.16
		Belgium	Lier	LUCIL064-12	22	23	100	100*	100	99.23
		France	Le Soler	LUCIL065-12	22	24	100	100*	100	99.29
		France	Le Soler	LUCIL066-12	23	25	99.86	100*	100	99.23
		Belgium		LUCIL067-12	22	24	100	100*	100	99.29
		Belgium	Auderghem/Oudergem	LUCIL068-12	24	26	100	100	100	99.23
		Belgium	Schaerbeek/Schaarbeek	LUCIL069-12	25	27	100	100*	100	99.16
		Belgium	Genk	LUCIL070-12	22	24	100	100*	100	99.29
		Belgium	Genk	LUCIL071-12	26	28	99.86	100*	100	99.23
		France		LUCIL072-12	22	29	100	100*	100	99.23
		France		LUCIL073-12	22	29	100	100*	100	99.23
		France		LUCIL074-12	22	29	100	100*	100	99.23
		France		LUCIL075-12	22	29	100	100*	100	na
		France		LUCIL076-12	22	29	100	100*	100	99.23
	*Protophormia terraenovae* (Robineau-Desvoidy, 1830)	Belgium	Andrimont	NICC056-13	27	na	99.86	100	99.7	na
		Belgium	Auderghem/Oudergem	NICC057-13	27	na	99.86	100	99.7	na
		Belgium	Andrimont	NICC058-13	28	na	100	100	99.85	na
		Belgium	Andrimont	NICC059-13	27	na	99.86	100	99.7	na
		Belgium	Andrimont	NICC060-13	27	na	99.86	100	99.7	na
		Belgium	Andrimont	NICC061-13	29	na	100	100	99.85	na
		Belgium	Andrimont	NICC062-13	28	na	100	100	99.85	na
		Belgium	Andrimont	NICC063-13	30	na	100	100	100	na
		Belgium	Andrimont	NICC064-13	28	na	100	100	99.85	na
		Belgium	Andrimont	NICC065-13	31	na	99.72	100	99.85	na
		Belgium	Andrimont	NICC066-13	28	na	100	100	99.85	na
		Belgium	Auderghem/Oudergem	NICC067-13	27	na	99.86	100	99.7	na
Fanniidae	*Fannia* sp1	Belgium	Soignes/Zoniën forest	NICC040-13	32	30		100		
	*Fannia* sp2	Belgium	Soignes/Zoniën forest	NICC041-13	33	31		100		
	*Fannia* sp3	Belgium	Soignes/Zoniën forest	NICC042-13	34	32				
Muscidae	*Eudasyphora cyanella* (Meigen, 1826)	Belgium	Soignes/Zoniën forest	NICC039-13	35	33	100	100		
	*Musca autumnalis*<br/> De Geer, 1776	Belgium	Soignes/Zoniën forest	NICC043-13	36	34	100	100		
	*Muscina levida* (Harris, 1780)	Belgium	Pecq	NICC044-13	37	35	100	100	100	
		Belgium	Pecq	NICC045-13	38	36	99.85	100	99.85	
		Belgium	Pecq	NICC046-13	37	37	100	100	100	
		Belgium	Pecq	NICC047-13	37	35	100	100	100	
		Belgium	Soignes/Zoniën forest	NICC048-13	37	38	100	100	100	
	*Muscina prolapsa* (Harris, 1780)	Belgium	Soignes/Zoniën forest	NICC049-13	39	39		99.85		
		Belgium	Soignes/Zoniën forest	NICC050-13	39	na		99.85		
		Belgium	Saint–Gilles/Sint–Gillis	NICC051-13	40	40		100		
		Belgium	Saint–Gilles/Sint–Gillis	NICC052-13	40	40		100		
		Belgium	Soignes/Zoniën forest	NICC053-13	40	na		100		na
	*Neomyia cornicina* (Fabricius, 1781)	Belgium	Soignes/Zoniën forest	NICC054-13	41	41				
	*Polietes lardarius* (Fabricius, 1781)	Belgium	Soignes/Zoniën forest	NICC055-13	42	42	99.84	99.85		

### Laboratory protocols

We extracted genomic DNA from one or two legs per specimen using the NucleoSpin Tissue Kit (Macherey-Nagel) and a final elution volume of 70 µl. Fragments of the COI marker were amplified using two primer pairs TY-J-1460/C1-N-2191 and C1-J-2183/TL2-N-3014 (Sperling et al. 1994, Wells and Sperling 1999). The fragment obtained with the first primer pair encompasses the barcode region of ca. 650 bp used for animals (Hebert et al. 2003). The assembly of the fragments obtained with both primer pairs generated a sequence of 1534 bp corresponding to the complete COI gene. Each 25 µl PCR reaction contained final concentrations of 0.2 mM dNTPs, 0.4 μM of each primer, 2.0 mM MgCl_2_, 0.5 U of Taq DNA polymerase (Platinum, Invitrogen), 1 × PCR buffer and 2-4 µl DNA template. The thermal cycler program consisted of an initial denaturation step of 4 min at 94 °C, followed by 40 cycles of 30 s at 94 °C, 30 s at 45 °C and 90 s at 72 °C; with a final extension of 7 min at 72 °C. We cleaned PCR products using the NucleoFast96 PCR Kit (Macherey-Nagel) and sequenced them bidirectionally on an ABI 3130 Genetic Analyzer (Applied Biosystems) using the BigDye Terminator Cycle Sequencing Kit v3.1.

### Sequence quality control and analysis

We assembled and aligned sequences in SeqScape v2.5 (Applied Biosystems) and confirmed the absence of stop codons using MEGA5 (Tamura et al. 2011). Sequences were deposited in BOLD (BOLD process ID’s are given in Table 1) and GenBank. All different haplotypes were extracted from the aligned sequences using the R package PEGAS (Paradis 2010). We calculated pairwise p-distances (i.e. the proportion of sites at which two sequences differ) and searched for haplotypes that were shared among species.

Haplotypes were then used as queries to search for most similar sequences in two public databases: GenBank (NCBI, National Centre for Biotechnology) and BOLD (the Barcode of Life Data Systems). These most similar sequences will be called “best matches” *sensu* Meier et al. (2006) in the following. In GenBank, searches were done using MegaBLAST, the Basic Local Alignment Search Tool (BLAST) optimised for highly similar sequences (Zhang et al. 2000, Morgulis et al. 2008). In BOLD, the in-built Identification System (IDS) was applied (Ratnasingham and Hebert 2007) on two different databases: the Public Record Barcode Database (341 580 sequences; 45 368 nominal species and 11 732 interim species, or candidate species that have not been described yet on 24 May 2013) and the Species Level Barcode Records (1 367 662 sequences; 127 679 species and 53 394 interim species on 24 May 2013). The first database comprises the same records as GenBank because both libraries regularly synchronize their published records. In BOLD, this database of public records is a collection of COI records of minimum 500 bp from the published projects of BOLD. The Species Level Barcode Records of BOLD is used by default in IDS. It contains, in addition to the published COI records, early data release of COI records with a species level identification and a minimum sequence length of 500 bp. These early releases contain all information necessary for barcodes (locality and date of sample collection, trace files and sequence information as well as voucher specimen and database identifiers), have passed computerized quality checks of BOLD but might include provisional taxonomic assignments (Hebert et al. 2010).

In total, we applied five search strategies by submitting the barcode sequences to 1) GenBank, 2) the Public Records of BOLD, 3) the Species Level Records of BOLD including early releases, as well as 4) by using the barcode sequences as queries in combination with a keyword, “barcode”, in GenBank, and 5) by submitting COI sequences longer than the barcode fragment (1412–1534 bp) to GenBank. The use of the keyword “barcode” allowed us to filter the GenBank reference sequences and obtain only best matches that are tagged as barcodes, not only in the field “keyword” but also in any field of GenBank records. Longer COI sequences have not been submitted to BOLD because BOLD was developed to accept sequences from the strict barcode region only. In BOLD, IDS returns a list of maximum 99 best matches and provides a species-level identification for best close matches showing less than 1% divergence (Ratnasingham and Hebert 2007). Since BLAST searches are based on approximate alignments (regions of local similarity between sequences), species assignments are usually preferably performed on the basis of local alignments. Hence we verified that the best hits and their percentages of sequence identity obtained from the MegaBLAST searches in GenBank were identical to those (= 1–p-distance) calculated with MEGA5 (Tamura et al. 2011) using local databases downloaded from GenBank and aligned with CLUSTAL W (Thompson et al. 1994). Identifications were made on the basis of the highly similar best matches (> 99% similarity), according to the “best close match” method of Meier et al. (2006). We qualified each best match with a similarity of > 99% as correct if it had the same species name as the query or as incorrect if it had a different species name than the query. In addition, the identification of a query was considered as unambiguous if all best matches with a similarity of > 99% had the same species name. If this was not the case, then the identification was ambiguous. For each identification, we made sure that best close matches included only records properly identified to the species level by excluding the few records with provisional identifications (a code instead of a nominal species name). We also verified whether the alignment of the query with each best match comprised at least 600 bp. When no best match of > 99% similarity was retrieved for a given query, the presence of conspecific and congeneric barcode sequences of > 500 bp was investigated in both public libraries. If present, their divergences (p-distances) with the queries were calculated using MEGA5 (Tamura et al. 2011).

## Results

In total 85 sequences were obtained with more than 641 bp of the COI DNA barcode fragment, representing 42 haplotypes. The majority of them (63 sequences) involved a longer COI fragment (1412–1534 bp), representing 42 other haplotypes. Pairwise intraspecific p-distances ranged from zero to 0.5% and none of the species represented in this dataset shared haplotypes.

### Search procedures 1 and 2

**Figure 1.**
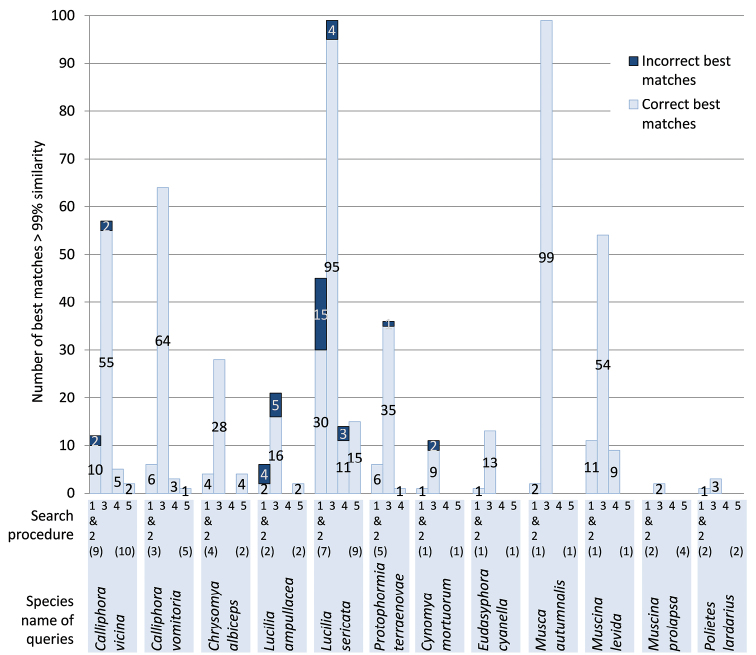
Best matches obtained for each species using five different search procedures:Barcode fragment (642–658 bp) submitted to GenBank (1) and the public records of BOLD (2); barcode fragment submitted to the species level records of BOLD, including early-released sequences (3); barcode fragment and keyword “barcode” submitted to GenBank (4) and longer COI fragment (1412–1534 bp) submitted to GenBank (5). Numbers of haplotypes used as queries are between parentheses. Longer COI fragments were obtained for all species except for *Protophormia terraenovae*.

Using the 42 haplotypes of the barcode region as a query yielded the same results in GenBank and in the Public Record Barcode Database of BOLD. Best matches of > 99% similarity were retrieved for 36/42 haplotypes, representing 11 out of 16 species (Table 1). These best matches were either identical (17/36) or differed from the query in less than three substitutions (19/36). We obtained at least one correct best match for each query. However, species identifications were either unambiguous (18 queries, 8 species) or ambiguous (18 queries, 3 species). For two queries, best matches included species of another genus: *Musca domestica* Linnaeus, 1758was found for *Calliphora vicina* and *Chrysomya megacephala* (Fabricius, 1794) for *Lucilia ampullacea*. In all other cases of ambiguous identification, best matches involved congenerics: *Calliphora croceipalpis* Jaennicke, 1867 was found for *Calliphora vicina*, *Lucilia cuprina* (Wiedemann, 1830) for *Lucilia sericata* and *Lucilia porphyrina* (Walker, 1856) for *Lucilia ampullacea*. Finally, the number of best matches with > 99% similarity varied from one to more than 99 per query (the number of best matches displayed by BOLD is limited to 99). For five species, less than five sequences with a similarity of > 99% were retrieved (Figure 1).

For six queries, the best matching similarities were < 93.5%. These included the haplotypes of *Fannia* sp1, sp2 and sp3, *Muscina prolapsa* and *Neomyia cornicina*. There were no COI sequences of *Muscina prolapsa* or of *Neomyia cornicina* in GenBank. For *Fannia*, fragments of the barcode region of > 500 bp were available for 14 specimens representing four species, viz. *Fannia canicularis* (Linnaeus, 1761), *Fannia scalaris* (Fabricius, 1794), *Fannia brevicauda* Chillcott, 1961 and *Fannia serena* (Fallen, 1825) but their p-distances with our three *Fannia* haplotypes ranged from 6.6% to 16.2%.

### Search procedure 3

Using the Species Level Barcode Records dataset of BOLD (Table 1), highly similar best matches (> 99%) were retrieved for 40/42 queries (14/16 species). Correct best matches were retrieved for all specimens identified at the species level, but identifications were often ambiguous (25 queries, 6 species). This method yielded a higher proportion of best matches of > 99% similarity than when the search was restricted to public records (95% of the queries instead of 86%). However, the proportion of unambiguous identifications was smaller (38% instead of 50% of the queries; Table 2). Yet, in contrast to all the other searches, early-released sequences provided two correct matches for *Muscina prolapsa*, one match for *Fannia* sp2 and three matches for *Fannia* sp1 (correct at the genus level). The latter identification was ambiguous since two best matches showed 100% similarity with *Fannia lustrator* (Harris, 1780) and one showed 99.85% similarity with *Fannia pallitibia* (Rondani, 1866). The two queries for which best matches were of < 99% similarity were from *Fannia* sp3 and *Neomyia cornicina*. No barcodes were available for *Neomyia cornicina* in BOLD.

**Table 2.** **Evaluation of the DNA-based identifications obtained in this study using five search procedures:** barcode fragment submitted to GenBank (1), to the public records of BOLD (2), to the species level records of BOLD including early releases (3), to the records of GenBank that are tagged as barcodes (4) and longer COI fragment submitted to GenBank (5). Only best matches of > 99% similarity were considered. OK: correct unambiguous identification; OK +: ambiguous identification due to correct and incorrect best matches (species names associated with incorrect best matches are given with the abbreviated genus name in case of congeneric matches); *: ambiguous identifications where the best correct and the best incorrect matches had the same similarity with the query; best correct matches were more similar to the query than best incorrect matches in all other ambiguous identifications; na: longer COI fragment not available; empty cell: no best match above 99% similarity. Numbers without parentheses were obtained with the barcode fragment and numbers between parentheses were obtained with the longer COI fragment. In order to allow comparisons between the results obtained with the barcode and the longer COI datasets, values obtained with the barcode fragment of the sequences for which the longer COI fragment was available are given between brackets.

Species	Number of haplotypes	Search procedure			
		1 & 2	3	4	5
***Calliphora vicina***	9 (10)	OK + Calliphora croceipalpis*, Musca domestica*	OK + Calliphora croceipalpis*, Musca domestica*	OK	OK
***Calliphora vomitoria***	3 (5)	OK	OK	OK	OK
***Chrysomya albiceps***	4 (2)	OK	OK		OK
***Lucilia ampullacea***	2 (2)	OK + *Lucilia porphyrina*, *Chrysomya megacephala*	OK + *Chrysomya megacephala*		OK
***Lucilia sericata***	7 (9)	OK + *Lucilia cuprina*	OK + Lucilia cuprina*	OK + *Lucilia cuprina*	OK + *Lucilia cuprina*
***Protophormia terraenovae***	5 (0)	OK	OK + *Protophormia uralensis*	OK	na
***Fannia* sp1**	1 (1)		*Fannia pallitibia*, *Fannia lustrator*		
***Fannia* sp2**	1 (1)		*Fannia manicata*		
***Fannia* sp3**	1 (1)				
***Cynomya mortuorum***	1 (1)	OK	OK + *Cynomya cadaverina*		
***Eudasyphora cyanella***	1 (1)	OK	OK		
***Musca autumnalis***	1 (1)	OK	OK		
***Muscina levida***	2 (4)	OK	OK	OK	
***Muscina prolapsa***	2 (2)		OK		
***Neomyia cornicina***	1 (1)				
***Polietes lardarius***	1 (1)	OK	OK		
**% of species with matches > 99% similarity**		**69**[67]	**88**[87]	**31**[27]	(33)
**% of queries with matches > 99% similarity**		**86**[86]	**95**[95]	**62**[67]	(67)
**% of species with unambiguous ID**		**73**[70]	**57**[62]	**80**[75]	(80)
**% of queries with unambiguous ID**		**50**[42]	**38**[42]	**73**[68]	(68)
**% of species with ambiguous ID**		**27**[30]	**43**[38]	**20**[25]	(20)
**% of queries with ambiguous ID**		**50**[58]	**62**[58]	**27**[32]	(32)

### Search procedure 4

When both the barcode sequences and the keyword “barcode” were used as queries in GenBank, we retrieved best matches of > 99% similarity for *Calliphora vicina*, *Calliphora vomitoria*, *Lucilia sericata*, *Protophormia terraenovae* and *Muscina levida* (Tables 1 and 2). All best matches of > 99% similarity were correct and provided unambiguous identifications except for *Lucilia sericata*, which matched with both correct and incorrect species names (*Lucilia cuprina* and *Lucilia sericata*).

### Search procedure 5

Haplotypes of longer COI fragments (1412–1534 bp) were also submitted to a MegaBLAST search on GenBank. Best matches of > 99% similarity were obtained for all haplotypes of *Calliphora vicina*, *Calliphora vomitoria*, *Chrysomya albiceps*, *Lucilia ampullacea* and *Lucilia sericata*. Like in the previous analysis, all best matches were correct and provided unambiguous identifications except for *Lucilia sericata* (best matches included *Lucilia sericata* and *Lucilia cuprina*).

## Discussion

### Towards a COI reference database for the forensically important dipterans in Western Europe

With this study we contributed to the establishment of a local COI reference library for fly species of forensic importance in Belgium and France. As such, we provide the first barcodes for *Muscina prolapsa* and *Neomyia cornicina*. We also extended the geographic coverage of barcodes of species which hitherto were only sampled from a limited number of localities, e.g. *Cynomya mortuorum* and *Polietes lardarius* were each represented by only one barcode sequence from the UK (Kutty et al. 2008). Similarly, barcodes of *Muscina levida* were until now only available for samples from Canada, Germany (Renaud et al. 2012) and the USA (Nakano and Honda, unpublished). Conversely, barcodes of the other species sampled here were obtained from no more than five European countries. Ideally, a reliable reference library should comprise a large sampling of sequences, not only representing the European dipteran species that are currently used in forensics (whose development times have been studied under different temperature conditions), but also those of potential forensic interest (occurring on carcasses but whose biology has been less studied) and all their close relatives. Currently, 13 species belonging to 10 genera are being used in forensic investigations (Marchenko 2001, Grassberger et al. 2002, Richards et al. 2009, Velásquez et al. 2013). Hence, the geographic coverage of GenBank and BOLD is still far from comprehensive. Yet, we did not observe intraspecific COI divergences of > 1% at COI, neither among specimens sequenced in this study nor between them and their conspecific best matches in the public libraries (intraspecific distances among GenBank sequences were not calculated here). This indicates that geographic coverage does not always have to be complete to allow correct species identification. Nonetheless, a more comprehensive reference library may comprise more haplotypes, allowing a better assessment of the risk of incorrect identifications (Meier et al. 2006). Indeed, an increased sampling can result in a more difficult distinction between some closely related species (Bergsten et al. 2012) and this has considerable importance for courts.

### Evaluation of the DNA-based identifications of forensically important flies in Belgium and France provided by GenBank and BOLD

For 86% of the barcode fragments used as queries, we retrieved highly similar conspecific sequences (> 99% similarity) from GenBank and BOLD. The more divergent best matches (< 99% similarity) obtained for the remaining 14% of the queries would have produced either incorrect (*Muscina prolapsa* and *Neomyia cornicina*) or doubtful identifications (*Fannia*) if all best matches were taken into account for identification. The better performance of the best close match method compared to the simple best match method has already been reported (e.g. Meier et al. 2006, Virgilio et al. 2010). However, even with the best close match method, our results revealed three issues that can hamper the DNA-based identification of forensically important flies in Belgium and France using GenBank or BOLD: These databases 1) do not include some fly species of forensic interest, 2) include sequences from misidentified specimens and 3) cannot always discriminate between closely related species. Below, we discuss these three issues in more detail.

### 1) Species not represented in the libraries

Our results showed that some fly species collected at Belgian crime scenes are not represented by COI records in GenBank and BOLD. *Muscina prolapsa*, for which no barcode sequence is present in GenBank,colonises carrion and buried remains (Gunn and Bird 2011, Prado e Castro et al. 2012). Also, the identification of *Fannia* species of forensic interest (Prado e Castro et al. 2012) is hampered by their limited representation in GenBank and BOLD. *Neomyia cornicina* is currently not used for PMI estimation but the availability of reference sequences of such species collected on crime scenes can decrease the risk of incorrect identification and help to characterize the entomofauna surrounding the crime scene (Amendt et al. 2007).

### 2) Sequences from misidentified specimens

Identifications based on the barcode fragment were ambiguous for 50% of the queries and for 27% of the species. Some ambiguous identifications can result from misidentified sequences in the libraries and could be corrected after re-examining the voucher specimens (Collins and Cruickshank 2013). In our study, the best matches with sequences from different genera could be the result of misidentifications: records of *Musca domestica* (GenBank accession number JQ350716) and *Chrysomya megacephala* (KC135926) matched our sequences of *Calliphora vicina* and *Lucilia ampullacea*, respectively.

### 3) Identification of closely related species

Still, most ambiguous identifications involved closely related species that are not necessarily incorrectly identified (Stevens et al. 2002, Sonet et al. 2012). For example, Wells et al. (2007) and Wells and Stevens (2008) showed that the barcodes of several specimens of *Lucilia cuprina* (from Hawaii and Asia) are more similar to those of *Lucilia sericata* than to those of other *Lucilia cuprina* specimens. This explains the ambiguous identification obtained here for *Lucilia sericata*. In some cases, the arbitrary similarity threshold, below which matches cannot be used for identification, is too low. Consequently, best close matches with conspecific and allospecific sequences are considered for identification, even if all conspecific best matches are closer to the query than any of the allospecific ones. To solve this problem, the similarity threshold can be adapted according to the gap between intra- and interspecific distances observed in this particular group of species (Lefébure et al. 2006, Collins and Cruickshank 2013, Puillandre et al. 2012, Virgilio et al. 2012). Here, we only used an arbitrary threshold of 99% similarity. A stricter similarity threshold (e.g. 99.5%) wouldresolve ambiguous identifications obtained for *Lucilia ampullacea*,for *Lucilia sericata* (but not when early releases of BOLD are used) and for *Cynomya mortuorum* (Tables 1 and 2).

Similarity values between the query and its best matches can be calculated using several methods. Here, similarities with GenBank records were determined as 1 - p-distances but no explicit information was found on the exact method used by the IDS of BOLD to determine the similarity values. Even if the IDS of BOLD applied a different method than ours, – distances are standardly corrected using the Kimura 2-parameter model (Kimura 1980) in DNA barcoding (Hebert et al. 2003) – the two searches (1 and 2) using the same queries against the same public records resulted in an identical list of highly similar best matches. Several studies have indeed observed that biases due to different distance calculation methods are less severe with similar sequences than with divergent ones (Collins et al. 2012, Fregin et al. 2012).

### Expanding or restricting the search in GenBank and BOLD?

It is striking that identifications provided by GenBank and BOLD for the barcode fragment were either ambiguous or involved a rather limited number of very similar reference sequences (Figure 1). Therefore, we tested alternative search strategies to optimise the number of best matches and minimise the number of ambiguous identifications. For this, we used different options offered by GenBank and BOLD by 1) including early releases from BOLD in the reference library, 2) adding the keyword “barcode” as a query in GenBank and 3) using longer COI sequences as queries in GenBank.

Including early releases as reference sequences in BOLD increased the number of best matches of > 99% similarity but also increased the proportion of ambiguous identifications (Table 1). Early releases might not have passed all controls that authors and reviewers make in the process of publication (e.g. Schindel et al. 2011). They are therefore more prone to errors. However, their early release allows the detection of errors and inconsistencies before publication, which is an efficient way to improve the quality of the reference libraries. In addition, they largely outnumber the published sequences and may include precious additional information such as rare haplotypes.

In order to improve the search for sequences that have been produced for DNA barcoding purposes, we added the word “barcode” to each query in GenBank. With this procedure, the number of best matches of > 99% similarity and the proportion of ambiguous matches drastically decreased. The same tendency was observed when longer COI sequences (1412–1534 bp) were used as queries. This is due to the smaller number of reference sequences that are tagged as barcodes or are longer than the standard barcode fragment. Therefore, this kind of search is currently only relevant for the identification of fly species of forensic interest that are well represented by longer COI reference sequences or that are tagged as barcodes. Moreover, longer DNA fragments are not always easy to sequence from degraded forensic samples (Mazzanti et al. 2010). Due to the limited number of best matches of > 99% similarity retrieved by these two options, it was not possible to assess their benefit when trying to minimise the proportion of ambiguous identifications.

## Conclusion

Even if BOLD and GenBank contain the same public records, they offer different options for optimizing their use as reference libraries. For barcode data, we recommend using the BOLD Identification System and searching the dataset including early-released sequences (Species Level Barcode Records). This option optimises the number of best-matches and allows to verify the quality of the data (published or early-released sequence, barcode compliant or not, link with voucher specimens, etc.). When working with reference material, we encourage the early release of the data and the correction of any mistake detected at this stage (e.g. misidentification). Furthermore, entering sequences into a BOLD project gives access to a workbench with supplementary tools (tables with best matches, best close matches and construction of Neighbour-Joining trees), that are useful for quality control (Ratnasingham and Hebert 2007). If ambiguous identifications are obtained, it is possible to restrict the search to the published sequences only (BOLD or GenBank) or to the sequences that were produced in the framework of the DNA barcoding initiative. Finally, a further validation with other DNA fragments, morphological characters or ecological evidence might be necessary. Without such a validation, identifications will remain questionable and can only be applied to more inclusive taxonomic levels (Wilson et al. 2011). Although DNA barcoding has been validated for forensic use (Dawnay et al. 2007), its applicability in forensics clearly depends on the reliability of the data and of the identification method used (Pereira et al. 2010, Linacre et al. 2011).
